# Spinach Extract Reduces Kidney Damage in Diabetic Rats by Impairing the AGEs/RAGE Axis

**DOI:** 10.3390/ijms26104730

**Published:** 2025-05-15

**Authors:** Javier Flores-Estrada, Agustina Cano-Martínez, Luz Ibarra-Lara, Adriana Jiménez, Carmen Palacios-Reyes, Luis J. Pinto García, María G. Ortiz-López, Olga Nelly Rodríguez-Peña, Luis Barbo Hernández-Portilla

**Affiliations:** 1 División de Investigación, Hospital Juárez de México, México City 07760, Mexico; adijh@hotmail.com (A.J.); ljpinto08@comunidad.unam.mx (L.J.P.G.); gortizl@prodigy.net.mx (M.G.O.-L.); 2 Departamento de Fisiología, Instituto Nacional de Cardiología Ignacio Chávez, México City 14080, Mexico; agustina.cano@cardiologia.org.mx; 3 Departamento de Farmacología, Instituto Nacional de Cardiología Ignacio Chávez, México City 14080, Mexico; luz.ibarra@cardiologia.org.mx; 4 Departamento de Ciencias Médicas, División de Ciencias de la Salud, Universidad de Guanajuato, Campus León, Guanajuato 37000, Mexico; cyapalacios@gmail.com; 5 Laboratorio de Biogeoquímica, Unidad de Biología, Tecnología y Prototipos (UBIPRO), Facultad de Estudios Superiores Iztacala, Universidad Nacional Autónoma de México, Av. de los Barrios No. 1, Tlalnepantla 54090, Mexico; onel.rodriguez@iztacala.unam.mx; 6 Laboratorio Nacional en Salud, Facultad de Estudios Superiores Iztacala, Universidad Nacional Autónoma de México, Av. de los Barrios No. 1, Tlalnepantla 54090, Mexico; lbarbo@unam.mx

**Keywords:** kidney, advanced glycation end products, Nε-(1-Carboxymethyl)-L-lysine, RAGE, inflammation, fibrosis, spinach

## Abstract

The interaction between advanced glycation end products (AGEs) and their RAGE receptor (AGEs/RAGE axis) triggers several signaling pathways that lead to the development of diabetic nephropathy (DN). One of the most studied AGEs is Nε-(1-Carboxymethyl)-L-lysine (CML). *Spinacia oleracea* is an edible plant with beneficial health properties, but its effect on the AGE/RAGE axis in kidney damage is unknown. Objective: We aimed to investigate the functional role of spinach methanolic extract (SME) on kidney damage in diabetic rats associated with the CML/RAGE axis. Methods: Forty adult male Wistar rats were used in this study and divided into four groups: control rats (CTRL), SME-administered CTRL (400 mg/kg; SME), streptozotocin-induced diabetic nephropathy rats (STZ), and SME-treated STZ (STZ-SME); treatments were administered daily. After 12 weeks, serum AGEs, creatinine in urine, and lipid peroxidation in kidneys were measured. The distribution and expression levels of inflammatory and fibrotic mediators and RAGE signaling were evaluated through immunohistochemistry (NOX4, CML, RAGE, nuclear NF-κB, TNF-α, IL-1β, TGF-β1, SMAD2/3, CTGF, and a-SMA) and immunolocalization of CML/RAGE. Results: Glycoside flavonoid derivatives, such as patuletin and spinacetin, were primarily identified in the extract. Kidneys from the STZ group showed altered morphology, dead cells in the proximal tubules, and increased oxidative stress markers; notably, these effects were improved by SME treatment (STZ-SME). The STZ-SME group showed a lower staining intensity for CML and RAGE, which was associated with a decrease in the expression of inflammatory and fibrotic factors compared with the STZ group. In all groups, the distribution of these markers varied among proximal tubule, glomerular, and interstitial cells. Conclusions: SME treatment may help to prevent or delay kidney damage in diabetic rats by regulating inflammatory and fibrotic processes associated with the AGEs/RAGE pathway, a mechanism involved in the development of nephropathy.

## 1. Introduction

Diabetic kidney disease (DKD), including diabetic nephropathy (DN), is a common complication of diabetes mellitus (DM) and the leading cause of end-stage renal disease worldwide, driving the global demand for dialysis or kidney replacement therapies [1,2]. Approximately 50% of patients with type 2 diabetes mellitus (T2DM) and 33% of those with type 1 diabetes mellitus (T1DM) are estimated to develop chronic kidney disease during their lifetime [2,3]. DKD is characterized by mesangial cell injury, extracellular matrix accumulation, thickening of the glomerular and tubular basement membranes, and podocyte dysfunction, leading to proteinuria, glomerulosclerosis, tubular damage, and progressive decline in renal function, ultimately increasing morbidity and mortality [3,4].

While hypoglycemic and antihyperlipidemic agents, renin–angiotensin–aldosterone system (RAAS) inhibitors, sodium–glucose co-transporter-2 (SGLT2) inhibitors, and anti-inflammatory therapies are considered promising interventions [4,5], diabetic nephropathy progression cannot be halted entirely even with stringent glycemic and blood pressure control [6]. Therefore, further research is needed to elucidate the mechanisms underlying kidney injury and identify novel therapeutic strategies.

The formation and accumulation of advanced glycation end products (AGEs) play a pivotal role in the pathogenesis of DKD. These irreversible compounds, which are closely associated with hyperglycemia, result from non-enzymatic reactions between amino groups of proteins (lysine and arginine residues) and carbonyl groups of reducing sugars, leading to Schiff base and Amadori product formation. Under oxidative conditions, these intermediates evolve into reactive 1,2-dicarbonyl species, causing protein cross-linking and functional impairment—a process integral to the Maillard reaction [2,7]

Under physiological conditions, AGEs are eliminated through extracellular proteolysis and receptor-mediated endocytosis, followed by intracellular degradation in proximal tubules [8,9]. In diabetes, however, accumulation of AGEs promotes oxidative stress, growth factor release, and inflammatory mediator production, contributing to the clinical and morphological manifestations of DKD [3,10]. Nε-(1-Carboxymethyl)-L-lysine (CML) is one of the most abundant AGEs in the serum and tissues of diabetic patients and experimental models and has been strongly associated with glomerular sclerosis and tubular injury [10,11].

The interaction between AGEs and the receptor for AGEs (RAGE) exacerbates renal inflammation and fibrosis by activating downstream signaling pathways [11,12,13]. AGE-RAGE interaction increases transforming Growth Factor Beta 1 (TGF-β1) expression, promoting extracellular matrix deposition and thickening of the glomerular basement membrane [14]. This process also involves epithelial-to-mesenchymal transition and is associated with increased expression of proinflammatory cytokines, including tumor necrosis factor-alpha (TNF-α) and interleukin 1 beta (IL-1β) [15,16].

Promising anti-glycation strategies include cross-link breakers, redox-active metal ion chelators, free radical scavengers, carbonyl trappers, RAGE antagonists, antioxidants, and aldose reductase inhibitors [17]. However, the clinical translation of these agents remains limited by safety concerns, necessitating further investigation.

Phytochemicals derived from plant-based foods represent an attractive alternative due to their antiglycation properties and better safety profiles. Natural compounds such as berberine, chrysin, and garlic extracts have been reported to inhibit Maillard reactions and modulate the AGEs/RAGE axis [14].

Spinach (*Spinacia oleracea* L.) is a nutrient-rich leafy vegetable containing abundant fiber, minerals, vitamins, and polyphenols, particularly flavonoids such as patuletin, spinacetin derivatives, and smaller amounts of quercetin and kaempferol [18,19,20,21]. In vitro, spinach extracts and related phytochemicals have shown potent inhibition of the formation of AGEs and neuroprotection against oxidative stress by suppressing methylglyoxal (MGO) production, reducing RAGE expression, and attenuating proinflammatory and apoptotic factors [22,23,24,25]. However, their potential role in preventing kidney damage through modulation of the AGEs/RAGE axis remains unexplored.

Given the critical role of AGEs and RAGE in DKD progression, there is an urgent need to develop new phytochemical-based strategies targeting this axis. Therefore, this study investigated the protective effects of spinach methanolic extract (SME) against kidney injury linked to the AGEs/RAGE pathway in streptozotocin-induced diabetic rats.

## 2. Results

### 2.1. Flavonoid Characterization in SME

**Figure 1:**
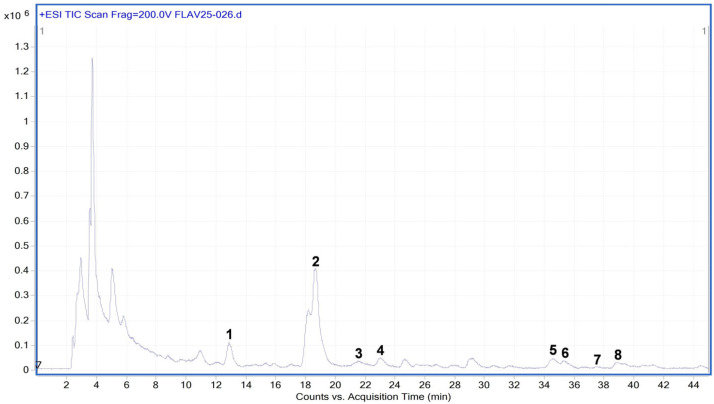
Total ion chromatogram of flavonoids in *S. oleracea* analyzed using HPLC-ESI-TOF-MS. (1) patuletin-3-O-β-d-glucopyranosyl-(1→6)-[β-d-apiofuranosyl-(1→2)]-β-d-glucopyranoside; (2) spinacetin 3-O-glucosyl-(1→6)-[apiosyl(1→2)]-glucoside; (13) patuletin-3-O-(2″-coumaroylglucosyl)-(1→6)-[apiosyl-(1→2)]-β-D-glucopyranoside; (4) spinacetin-3-O-glucosyl-(1→6)-glucoside; (5) spinacetin-3-O-(2″-coumaroylglucosyl)-(1→6)-β-D-glucopyranoside; (6) spinacetin 3-O-(2″-feruloylglucosyl)-(1→6)-β-D glucopyranoside; (7) spinatoside; (8) jaceidin 4′-glucuronide. Unidentified peaks: 1–6, 9, 12, 13.

**Figure 2:**
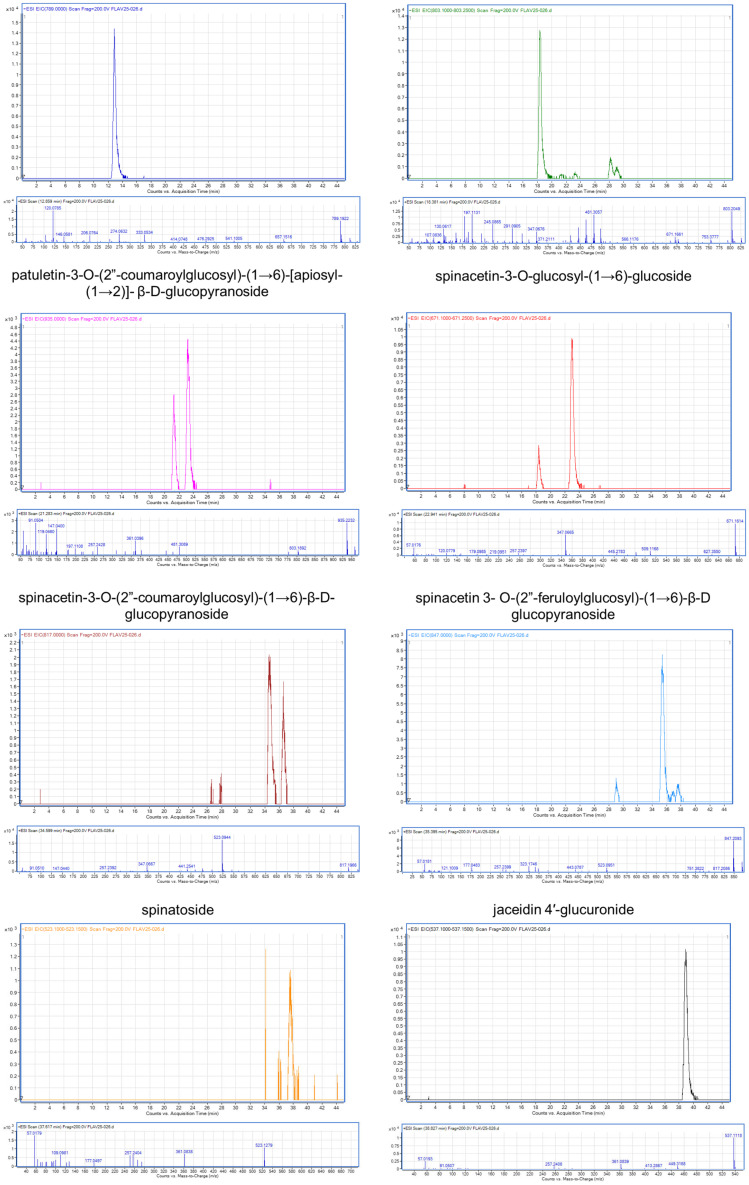
Chromatogram of the extracted ion and mass spectra of identified flavonoids analyzed using HPLC-ESI-TOF-MS.

The HPLC-ESI-TOF-MS analysis showed the presence of eight flavonoids (Figure 1) identified as follows: patuletin-3-O-β-d-glucopyranosyl-(1→6)-[β-d-apiofuranosyl-(1→2)]-β-d-glucopyranoside, spinacetin-3-O-glucosyl-(1→6)-[apiosyl-(1→2)]-β-D-glucopyranoside, patuletin-3-O-(2″-coumaroylglucosyl)-(1→6)-[apiosyl-(1→2)]-β-D-glucopyranoside, spinacetin-3-O-glucosyl-(1→6)-glucoside, spinacetin-3-O-(2″-coumaroylglucosyl)-(1→6)-β-D-glucopyranoside, spinacetin 3-O-(2″-feruloylglucosyl)-(1→6)-β-D glucopyranoside, spinatoside, and jaceidin 4′-glucuronide (Table 1; Figure 2).

### 2.2. Effect of SME on STZ-Induced Changes in Body Weight, Glucose and Creatinine Levels, and Renal Pathological Features

**Figure 3:**
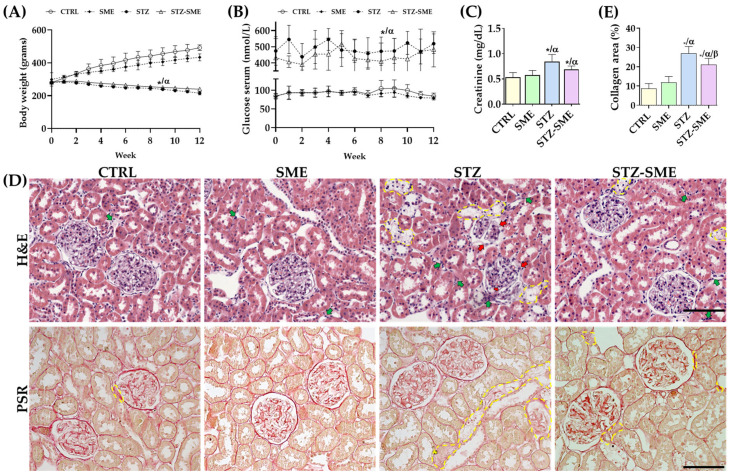
Effect of SME on STZ-induced kidney damage in rats. (**A**,**B**) Weekly body weight and glucose level measurements in the CTRL, SME, STZ, and STZ-SME groups. (**A**,**B**) Weekly body weight and glucose level measurements in the CTRL, SME, STZ, and STZ-SME groups (**C**) Serum creatinine levels after 12 weeks. (**D**) Representative histological images of H&E staining and picrosirius red (PSR) staining of renal tissue sections. (**E**) percentage of collagen fibers measured by PSR staining. Yellow dashed lines indicate tubular epithelial cell necrosis (H&E) and collagen accumulation (PSR). Green arrows point to inflammatory infiltrates, while red arrows highlight glomerular injury, characterized by glomerular tuft contraction (*), mesangial disorganization, and thickening of the glomerular capsule. Data are expressed as the mean ± SD (*n* = 8); * *p* < 0.05 vs. CTRL; α *p* < 0.05 vs. SME; and β *p* < 0.05 vs. STZ. Magnification: 200×. Scale bar = 100 μm.

During the 12-week experimental period, the STZ and STZ-SME groups exhibited significant hyperglycemia and lower body weight than the control (CTRL) and SME groups (*p* = 0.0001); Figure 3A,B). However, serum creatinine levels were significantly lower in the STZ-SME group than in the STZ group (*p* = 0.047), with no significant differences observed between the CTRL and SME groups (Figure 3C).

Histological analysis revealed greater renal damage in the STZ group compared with the STZ-SME group. Hematoxylin-eosin (H&E) staining demonstrated moderate tubular epithelial cell necrosis in the STZ group, primarily affecting the proximal tubules and characterized by brush border loss, epithelial flattening, and basement membrane thickening. In the STZ-SME group, a slight improvement was observed, whereas the control groups showed no histological abnormalities.

Regarding glomerular architecture, the STZ group displayed glomerular contraction, vascular congestion, cell swelling, and moderate interstitial inflammatory infiltration. In contrast, the STZ-SME group exhibited mild-to-moderate alterations in tubular and glomerular regions and mild interstitial inflammation. Nevertheless, histopathological changes in the STZ-SME group were still more pronounced than those observed in the CTRL and SME groups, which exhibited no detectable damage (Figure 3D, upper panel).

Kidney fibrosis was assessed by quantifying collagen fiber deposition using picrosirius red (PSR) staining (Figure 3D, bottom panel). The STZ group showed a significant percentage of collagen fibers, particularly around the tubules and glomeruli, compared with the STZ-SME group (*p* = 0.013) and the non-diabetic group (both *p* = 0.001). No significant differences were found between the CTRL and SME groups (Figure 3E). These findings suggest that hyperglycemia directly contributes to kidney injury and that SME treatment exerts anti-inflammatory and anti-fibrotic effects, thereby improving renal histopathology.

### 2.3. Effect of SME on STZ-Induced Nephrotoxicity

To evaluate whether SME treatment reduces STZ-induced nephrotoxicity, we assessed oxidative stress by measuring malondialdehyde (MDA) levels, nicotinamide adenine dinucleotide phosphate oxidase 4 (NOX4) intensity staining, and the percentage of cell death indicated by caspase 3 (casp-3) staining.

**Figure 4:**
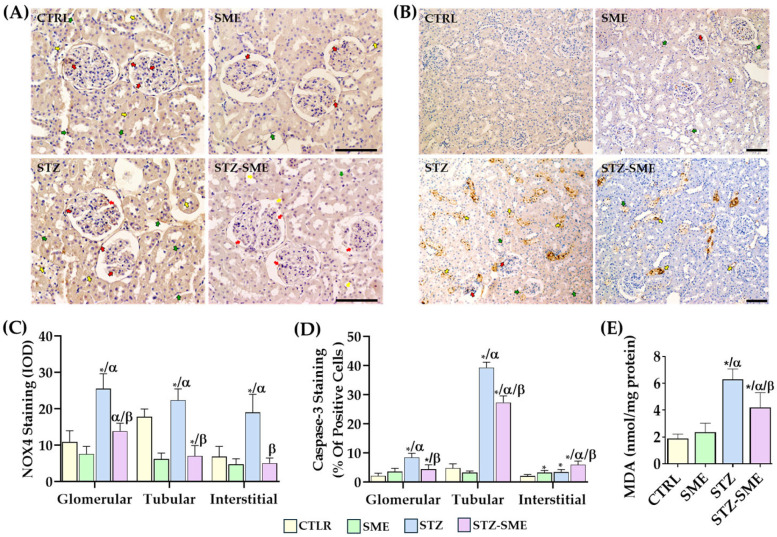
Effect of SME on STZ-induced nephrotoxicity. (**A**,**B**) Representative images showing the localization of NOX4 (magnification 100×) and casp-3 staining (magnification 200×) in the CTRL, SME, STZ, and STZ-SME groups. (**C**) Integrated optical density (IOD) for NOX4, (**D**) percentage of positive cells for casp-3 staining, and (**E**) MDA levels in kidney tissue. Red, yellow, and green arrows indicate positively stained glomerular, tubular, and interstitial cells, respectively. * *p* < 0.05 vs. CTRL; α *p* < 0.05 vs. SME; β *p* < 0.05 vs. STZ. Data are presented as the mean ± SD (*n* = 8). Scale bar = 100 μm.

In the STZ-SME group, levels of MDA and staining for NOX4 and casp-3 were significantly lower than in the STZ group (*p* < 0.05). NOX4 staining was broadly distributed throughout the renal cortex (Figure 4A), whereas casp-3 staining was primarily localized to the nuclei and cytoplasm of proximal tubular cells (Figure 4B). In the STZ group, NOX4 staining intensity, casp-3-positive cells, and MDA levels were significantly higher compared with the STZ-SME, SME, and CTRL groups (*p* < 0.01) (Figure 4B–E, respectively). In the CTRL and SME groups, compared with the STZ-SME group, both NOX4 staining intensity and the percentage of casp-3-positive cells were significantly lower in the glomerular, tubular, and interstitial regions (*p* < 0.01). In some cases, both proteins showed a decreasing trend without reaching statistical significance. No significant differences were detected between the CTRL and SME groups, except for a higher percentage of casp-3-positive cells in the interstitial region of the SME group (*p* = 0.043) (Figure 4C,D).

### 2.4. The Effect of SME on the Accumulation of CML and the Expression of RAGE

**Figure 5:**
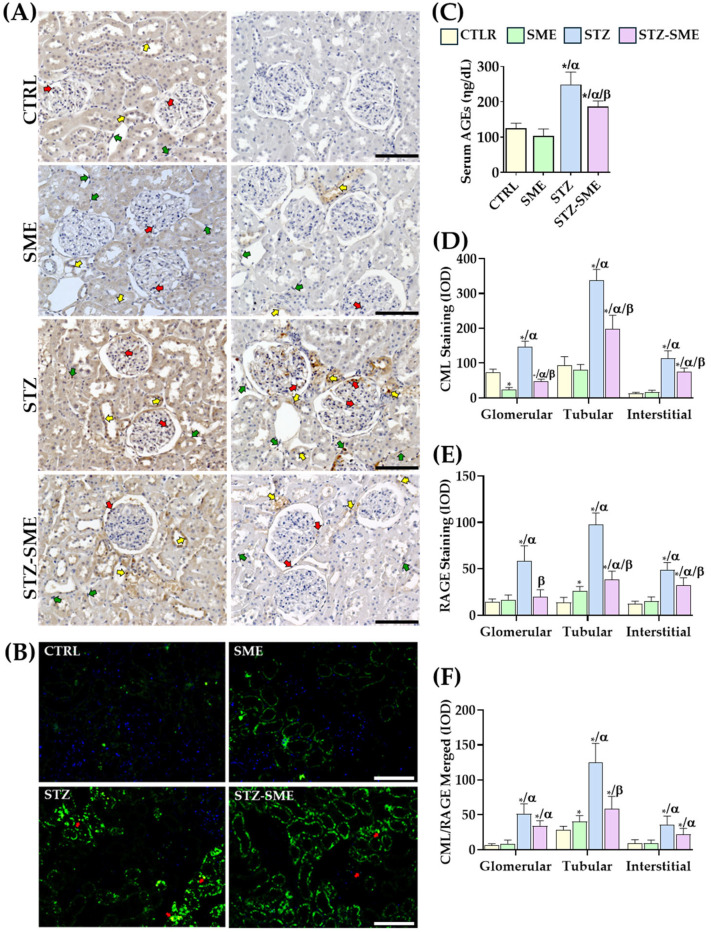
Effect of SME on the levels of CML-modified proteins and RAGE expression in the kidney cortex. (**A**) Total serum levels of advanced glycation end-products (AGEs) in the CTRL, SME, STZ, and STZ-SME groups. (**B**) Distribution of CML and RAGE in kidney tissue. (**C**) serum levels of AGEs (ng/dL), (**D**,**E**) IOD measurements for CML and RAGE immunohistochemistry. (**E**) Fluorescence colocalization images of CML and RAGE. (**F**) IOD values of CML/RAGE colocalization. Red, yellow, and green arrows indicate positively stained tubular, glomerular, and interstitial cells, respectively. Data are expressed as the mean ± SD (*n* = 8). * *p* < 0.05 vs. CTRL, α *p* < 0.05 vs. SME, and β *p* < 0.05 vs. STZ. Magnification: 200×. Scale bar = 100 μm.

The levels of total serum AGEs in diabetes are influenced by sustained hyperglycemia. To investigate the anti-glycation effect of SME in diabetic rats, serum levels of total AGEs and CML accumulation in the renal cortex were evaluated. As shown in Figure 5C, SME treatment significantly reduced serum AGE levels compared with the untreated STZ group (*p* = 0.032), although AGE levels in the STZ-SME group remained higher than those in the CTRL and SME groups (*p* < 0.01). No significant differences were observed between the CTRL and SME groups.

The distribution and accumulation of CML in the renal cortex were assessed by staining intensity (Figure 5A,D). CML staining was localized in the nuclei and cytoplasm of tubular, glomerular, and interstitial cells. Staining intensity was significantly higher in the STZ group compared with the STZ-SME group (*p* = 0.0028, *p* = 0.011, and *p* = 0.026, respectively). In CTRL kidneys, CML was distributed mainly in the cytoplasm of tubular cells with some positive nuclei. Notably, the SME group exhibited significantly lower CML staining intensity in glomerular cells than in the other groups (*p* < 0.01).

RAGE, a primary receptor for CML, plays a critical role in mediating cellular responses that activate inflammatory signaling pathways and promote fibrosis. Immunohistochemical analysis revealed that RAGE staining intensity in glomerular, interstitial, and renal proximal tubule cells was significantly higher in the STZ group than in the CTRL, SME, and STZ-SME groups (all *p* < 0.001), except in interstitial cells of the STZ-SME group (*p* = 0.042). Within the STZ-SME group, RAGE staining intensity in the renal proximal tubule and interstitial cells remained higher than in the CTRL and SME groups (*p* < 0.05). Significant differences between the CTRL and SME groups were detected only in tubular cells (*p* = 0.027) (Figure 5E).

CML/RAGE colocalization was evaluated through immunofluorescence (Figure 5B,F). Diabetic rats treated with SME exhibited reduced CML/RAGE staining intensity in renal tubular cells compared with the STZ group (*p* = 0.0039). Although a reduction in staining intensity was also observed in glomerular and interstitial cells, these differences were not statistically significant. Furthermore, CML/RAGE staining intensity was higher in the STZ-SME group than in the CTRL and SME groups (*p* < 0.05). Significant differences between the CTRL and SME groups were detected only in tubular cells (*p* = 0.046).

### 2.5. The Effect of SME on the Activation of the Inflammatory Response

**Figure 6:**
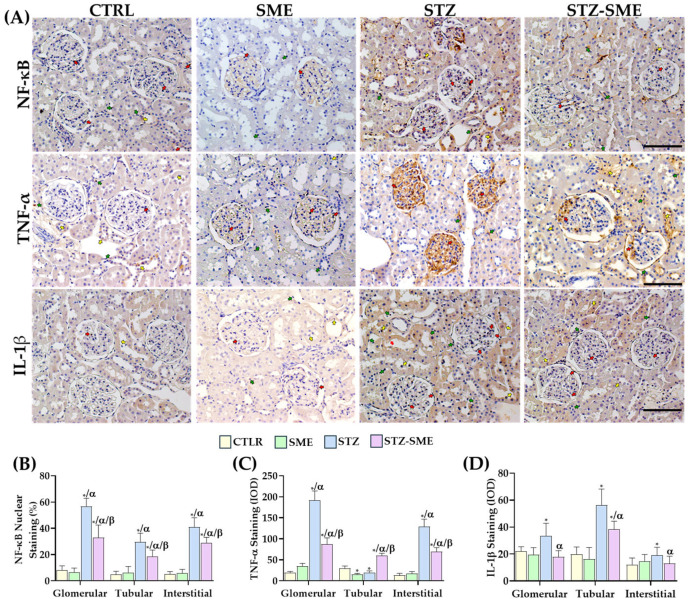
Effect of SME on STZ-induced kidney inflammation. (**A**) Representative images showing the distribution of nuclear NF-κB, TNF-α, and IL-1β in the kidney cortex of the CTRL, SME, STZ, and STZ-SME groups. Red, yellow, and green arrows indicate positively stained glomerular, tubular, and interstitial cells, respectively. (**B**) Percentage of cells positively stained for nuclear NF-κB, and (**C**) IOD measurements for TNF-α and (**D**) IL-1β. Data are expressed as the mean ± SD (*n* = 8). * *p* < 0.05 vs. CTRL, α *p* < 0.05 vs. SME, and β *p* < 0.05 vs. STZ. Magnification: 200×. Scale bar = 100 μm.

Since SME reduces CML accumulation, RAGE expression, and their interaction, the effect of SME on the activation of the inflammatory responses, specifically through the nuclear translocation of nuclear factor kappa B (NF-κB) nuclear translocation and TNF-α and IL-1β staining intensity in the renal cortex, was evaluated. Immunohistochemical assays were conducted to assess the distribution and percentage of NF-κB-positive nuclei staining. In the STZ-SME group, NF-κB nuclear staining was detected in glomerular, tubular, and interstitial cells. In contrast, in the other groups, staining was predominantly localized to glomerular and interstitial cells (Figure 6A). In the STZ group, the percentage of NF-κB-positive nuclei in both cell types was significantly higher compared with the STZ-SME (*p* < 0.01), CTRL (*p* < 0.0001), and SME (*p* < 0.0001) groups. Furthermore, the percentage in the STZ-SME group was significantly higher than that observed in the CTRL and SME groups. However, no significant difference was found between the CTRL and SME groups (Figure 6B).

TNF-α staining was predominantly observed in glomerular and interstitial cells, whereas IL-1β staining was diffusely localized in renal tubular cells (Figure 6A). TNF-α staining intensity was lower in the STZ-SME group compared with the STZ group (*p* < 0.0001) but remained higher than that in the CTRL and SME groups (*p* < 0.0001). However, in tubular cells, TNF-α staining intensity was higher in the STZ-SME group than in the STZ group (*p* = 0.00071). No significant differences were observed between the CTRL and SME groups (Figure 6C).

Regarding IL-1β staining intensity, a significant increase was observed in both glomerular (*p* = 0.039) and tubular cells (*p* = 0.014) in the STZ group compared with the STZ-SME group. Differences in IL-1β staining intensity among the STZ-SME, CTRL, and SME groups were significant only in tubular cells (*p* < 0.01). No significant difference was detected between the CTRL and SME groups (Figure 6D).

These findings suggest that treatment with SME may help reduce renal damage by regulating NF-κB nuclear translocation, thereby decreasing the expression of inflammatory cytokines.

### 2.6. Effect of SME on the Expression of Fibrotic Factors in the Kidney Cortex

**Figure 7:**
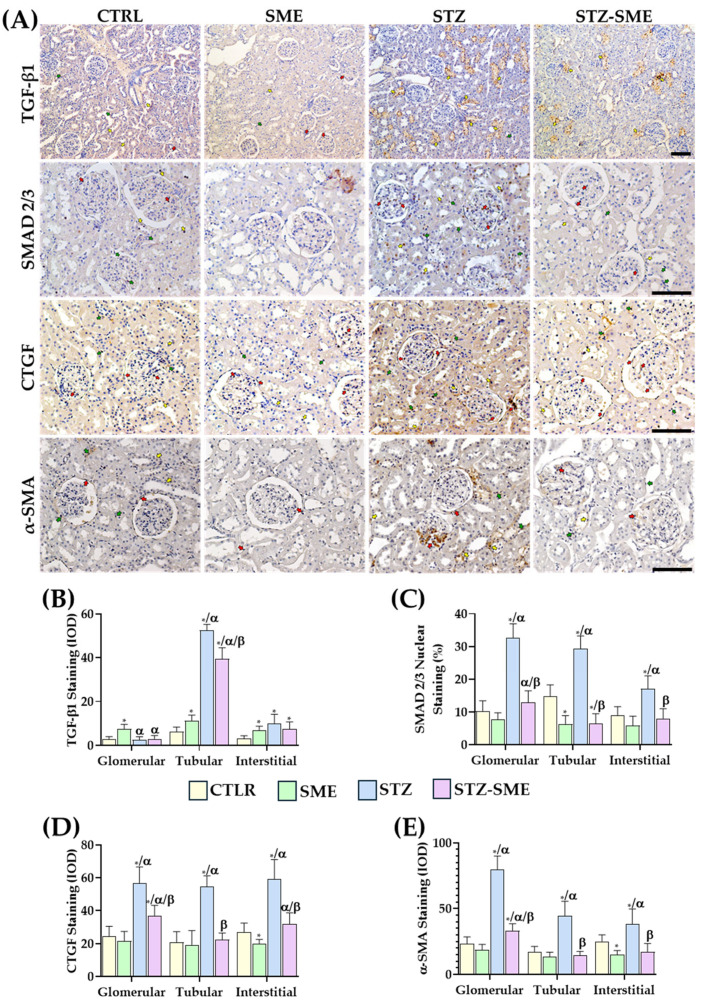
Effect of SME on STZ-induced kidney fibrosis. (**A**) Representative images showing the localization of TGF-β1, SMAD 2/3, CTGF, and α-SMA staining in the kidney cortex of the CTRL, SME, STZ, and STZ-SME groups. Red, yellow, and green arrows indicate positively stained glomerular, tubular, and interstitial cells, respectively. (**B**) IOD measurements for TGF-β1 (Magnification: 100×), (**C**) α-SMA, (**D**) CTGF, and (**E**) percentage of cells positively stained for nuclear SMAD2/3 (Magnification: 200×). Data are expressed as the mean ± SD (*n* = 8). * *p* < 0.05 vs. CTRL, α *p* < 0.05 vs. SME, and β *p* < 0.05 vs. STZ. Scale bar = 100 μm.

The effect of SME treatment on the expression of fibrotic markers, including TGF-β1, SMAD2/3 (small mother against decapentaplegic 2/3), CTGF (connective tissue growth factor), and α-SMA (alpha-smooth muscle actin), was evaluated in the kidneys of diabetic rats. As shown in Figure 7A, TGF-β1 staining was predominantly localized to the tubular region, with staining intensity significantly lower in the STZ-SME group compared with the STZ group (*p* = 0.014). Furthermore, TGF-β1 expression was markedly reduced in the CTRL and SME groups compared with STZ and STZ-SME (*p* < 0.0001). No significant differences in staining intensity were found between the STZ, STZ-SME, and SME groups in glomerular and interstitial cells. However, the SME group exhibited higher TGF-β1 staining intensity compared with the CTRL group (*p* < 0.01) (Figure 7B).

In renal tissue, SMAD2/3 immunoreactivity was detected in glomerular, tubular, and interstitial cell nuclei (Figure 7A). In the STZ group, the percentage of SMAD2/3-positive nuclei was significantly higher in these cell types compared with the CTRL, SME, and STZ-SME groups (all *p* < 0.01). In the STZ-SME group, the nuclear localization of SMAD2/3 exhibited a more complex pattern. Specifically, the percentage of SMAD2/3-positive nuclei in the glomerular region was higher than in the SME group (*p* = 0.019), although no significant difference was observed compared with the CTRL group. In contrast, in the tubular region, SMAD2/3 nuclear staining was lower in the STZ-SME group than in the CTRL group (*p* = 0.0059), with no significant difference compared with the SME group. No significant differences in the percentage of SMAD2/3-positive nuclei were found in interstitial cells across the STZ-SME, CTRL, and SME groups (Figure 7C).

In the STZ group, CTGF staining was distributed throughout the renal cortex (Figure 7A). The staining intensity in glomerular, tubular, and interstitial cells was significantly higher than in all other groups (*p* < 0.001). However, in the STZ-SME group, CTGF staining intensity in the glomerular region was higher compared with the CTRL and SME groups (*p* < 0.01), and an increased intensity was also observed in interstitial cells compared with the SME group (*p* = 0.0083). No significant differences were detected between the CTRL and SME groups (Figure 7D).

Similarly, α-SMA staining was distributed across renal cortex cells. Staining intensity in all cell types was significantly higher in the STZ group compared with the CTRL, SME, and STZ-SME groups (all *p* < 0.01). In the STZ-SME group, increased α-SMA staining intensity was observed only in glomerular cells compared with the CTRL and SME groups (*p* < 0.05). No significant differences were observed between the CTRL and SME groups (Figure 7E).

## 3. Discussion

Plant-derived phytochemicals have been proposed as protective agents against kidney damage associated with the accumulation of AGEs during diabetes. These compounds exert their effects by blocking interactions between reactive amino groups and reducing sugars, scavenging dicarbonyl species and hydroxyl radicals, and chelating trace metal ions that catalyze glycation, providing antioxidant benefits. Flavonoids such as resveratrol, kaempferol, and quercetin have been shown to inhibit oxidative stress and renal cytotoxicity induced by glyoxal (GlO), as well as prevent AGEs formation by scavenging methylglyoxal [22,28,29]. In this study, flavanol glycosides, including patuletin and spinacetin derivatives identified in SME, are suggested to contribute to its antiglycation activity. Research by Martha Gutierrez et al. [26] demonstrated that ten glucopyranoside flavonoids from methanolic spinach extract—prepared under conditions similar to those used in the present study—exhibited inhibitory activity against the formation of AGEs. Among them, patuletin-3-O-(2″-coumaroylglucosyl)-(1→6)-[apiosyl-(1→2)]-β-D-glucopyranoside dis-played the strongest antiglycation effect [26,27]. Moreover, previous studies have indicated that spinach extracts act as antioxidants and scavengers of dicarbonyl compounds [25]. Although compounds such as quercetin may contribute to this effect [22], the trace amounts present in SME under our extraction conditions likely explain why they were not detected [21].

This study evaluated the effect of SME on kidney damage induced by streptozotocin (STZ) in rats. STZ, a glucose analog, exerts diabetogenic effects by entering pancreatic β-cells via glucose transporter type 2 (GLUT2), promoting DNA damage, ROS production, mitochondrial dysfunction, and apoptosis [30]. STZ-induced glucotoxicity leads to dysfunction of renal proximal tubular epithelial cells, resulting in metabolic alterations characterized by the accumulation of AGEs and oxidative stress, thereby contributing to diabetic kidney disease. However, the direct nephrotoxic effects of STZ must also be considered. STZ causes DNA damage in renal tissue and activates the p53 signaling pathway in a dose-dependent manner; inhibition of p53 and GLUT2 has been shown to mitigate STZ-induced tubular injury [31]. Thus, in STZ-induced models, renal damage results from hyperglycemia and direct cytotoxicity.

H&E staining revealed that STZ administration in rats induced histological features such as early-stage necrosis of tubular cells, thickening of the tubular basement membrane, interstitial inflammation, and glomerular fibrosis. Furthermore, casp-3 staining indicated epithelial cell death in the proximal tubules, a marker of tubular degeneration. These findings suggest that STZ causes kidney damage through direct tubular cell injury and the elevation of blood glucose levels resulting from pancreatic dysfunction. Notably, SME administration significantly alleviated histological kidney damage, reducing fibrosis, inflammation, and tubular cell death.

Despite the presence of glucopyranoside flavonoids with known antiglycation and antioxidant properties, SME did not significantly lower blood glucose levels, which contrasts with the hypoglycemic effects of spinach leaf consumption or the administration of purified quercetin and kaempferol [21,32]. This discrepancy may be attributed to the low content of these polyphenolic compounds in the SME or to the irreversible damage of β cells induced by the STZ [30]. Nevertheless, the total levels of AGEs in serum were lower in the STZ-SME group compared with the STZ group, likely due to the scavenging of reactive carbonyl species and the inhibition of protein glycation. SME treatment also reduced the levels of oxidative stress markers, as shown by lower MDA levels and decreased NOX4 expression in renal tissues. These antioxidant effects are consistent with findings from other polyphenol-rich extracts, such as *Spatholobus suberectus*, which decreased CML accumulation and RAGE expression levels in diabetic models without affecting blood glucose or body weight [33,34].

CML is one of the major AGEs implicated in diabetic nephropathy, promoting glomerular sclerosis and tubular hypertrophy associated with lipid peroxidation [35,36,37,38,39]. Moreover, increased AGE deposition in diabetic kidneys may stem from STZ-induced tubular necrosis, impairing the glyoxalase (GLO-1 and GLO-2) system responsible for detoxifying reactive carbonyl compounds such as methylglyoxal and glyoxal, precursors to CML [8,38,40,41]. The reduction in CML accumulation in the STZ-SME group may reflect preserved glyoxalase activity due to decreased tubular apoptosis, supported by the lower percentage of casp-3-positive cells. Furthermore, SME may directly inhibit reactive carbonyl species formation, as previously demonstrated in vitro [25,28,42,43]. These findings suggest that SME exerts nephroprotective effects by reducing AGE deposition by trapping methylglyoxal (MGO) in the renal cortex. This mechanism may attenuate downstream signaling via RAGE, alleviating oxidative stress, inflammation, and tubular cell death.

The nephroprotective effect of SME appears to involve modulation of the CML/RAGE axis. Immunofluorescence revealed that CML/RAGE colocalization was significantly higher in diabetic kidneys but was markedly reduced following SME treatment. This suggests that SME may attenuate AGEs/RAGE-mediated signaling, thereby protecting renal structure and function. Additionally, RAGE distribution was broader in STZ kidneys, consistent with its multi-ligand binding capacity, including interactions with high mobility group box 1 (HMGB1) and S100 proteins [44,45,46,47,48,49]. Although CML levels are elevated in diabetic patients, the relative contribution of other RAGE ligands, such as high mobility group box 1 (HMGB1) and S100A8/A9, may also be essential factors [50].

Experimental studies have shown that plant-based extracts and phytochemical treatments provide nephroprotective effects by modulating the AGEs/RAGE axis [51,52,53,54,55,56]. Furthermore, advances in network pharmacology have unveiled novel strategies to identify the active components of medicinal plants, such as quercetin and kaempferol, that contribute to their pharmacological effects in diabetic nephropathy, particularly through the regulation of signaling pathways, including the AGEs/RAGE axis [57,58].

These effects are mediated by activating signaling pathways triggered by the AG-Es/RAGE interaction, such as RAGE/p38MAPK/NF-κB [29] and AGEs/RAGE/PKC-β/TGF-β1 [56]. In this context, the AGEs/RAGE/NOX4 pathway promotes the generation of reactive oxygen species (ROS) [55,59], which can contribute to renal complications, including tubular cell hypertrophy, podocyte apoptosis, inflammation, and fibrosis. It can be modulated by factors such as NF-κB [6,59,60,61]. Our results show significantly reduced NOX4 staining in the kidneys of diabetic rats treated with SME. In STZ-induced diabetic rats, NOX4 expression was observed in glomerular cells (potentially podocytes), interstitial cells, and tubular cells. Similar studies have demonstrated that loganin and catalpol protect against podocyte death in diabetic nephropathy by inhibiting the AGEs/RAGE/p38MAPK/p65NF-κB and AGEs/RAGE/NOX4 pathways [59]. These findings suggest that SME treatment may help prevent kidney damage by mitigating oxidative stress through the AGEs/RAGE/NOX4 signaling pathway.

The signaling pathway involving AGEs and RAGE generates reactive oxygen species (ROS) through the NOX4 pathway. This process triggers an inflammatory response by activating NF-κB, leading to the expression of TNF-α, IL-1β, and IL-6, which are critical in glomerular hypertrophy, podocyte injury, and tubulointerstitial fibrosis [62]. Therefore, the development of novel anti-inflammatory strategies is essential for nephroprotection. Flavonoids, such as patuletin, quercetin, and kaempferol—key components found in the leaves of Spinacia oleracea and certain members of the Kalanchoe family—have shown neuroprotective and gastroprotective effects in animal models by reducing oxidative stress and inflammation [25,63].

In this study, treatment with SME resulted in decreased NF-κB nuclear staining in kidney cortex cells, which was associated with a reduction in the expression of TNF-α and IL-1β. These findings suggest that these flavonoids may have a beneficial effect on STZ-induced kidney damage.

In the context of renal fibrosis, the AGEs/RAGE axis contributes to matrix accumulation, which is linked to increased TGF-β1 expression and activation of the canonical SMAD2/3 pathways, resulting in extracellular matrix deposition and glomerular basement membrane thickening [14,64]. Additionally, it promotes epithelial-to-mesenchymal and endothelial-to-mesenchymal transitions, marked by the upregulation of α-smooth muscle actin (αSMA) and connective tissue growth factor (CTGF), which are critical for the development of tubule-interstitial fibrosis [15,16]. Our study demonstrates that fibrogenesis in diabetic rats treated with SME was reduced, as evidenced by a significant decrease in TGF-β1 expression and reduced nuclear SMAD2/3 staining in renal tubular cells compared with untreated diabetic rats. The reduction in CTGF and αSMA staining levels suggests that SME prevents the transition of tubular epithelial cells into myofibroblasts. Histological analysis further supports this, showing reduced deposition of extracellular matrix proteins and fewer inflammatory infiltrates. These changes lead to a reduction in glomerular basement membrane thickening and tubular injury. Overall, the results indicate that spinach extract can mitigate the activation of the TGF-β1/SMAD2/αSMA and CTGF pathways, thus reducing renal fibrosis and preventing the development of diabetic kidney disease by inhibiting epithelial- and endothelial-to-mesenchymal transition.

## 4. Materials and Methods

### 4.1. Flavonoid Characterization

Fresh *S. oleracea* cultivated in an agricultural field during the fall–winter season was purchased at the Central de Abasto in Mexico City, Mexico. *S. oleracea* leaves were dehydrated at room temperature in the shade and ground. To remove non-polar compounds, 5 L of hexane was added per kilogram of dried spinach and refluxed for 3 h. The drying process involved the employment of a BUCHI Rotavapor^®^ R-215 (BÜCHI Labortechnik AG, Flawil, Switzerland). Subsequently, the polar components were extracted twice with 5 L of methanol at reflux for 3 h to achieve the highest bioactive concentration. To characterize flavonoid derivatives from *S. oleracea*, 20 mg of the extract was resuspended in water (1 mL) and centrifuged at 14,000 rpm for 3 min. The sample was analyzed through high-pressure liquid chromatography–electrospray–time of flight–mass spectrometry (HPLC-ESI-TOF-MS), for which a positive ion mode HPLC system was used, consisting of a vacuum degasser, autosampler, and binary pump (Infinity 1260, Agilent Technologies, Santa Clara, CA, USA), equipped with a Kinetex column (C-18 100A, 2.1 μm, 2.1 × 150 mm; Phenomenex, Torrance, CA, USA). The column temperature was maintained at 25 °C. The mobile phase consisted of HPLC-grade water (A) and HPLC-grade acetonitrile (ACN; B) with 0.1% formic acid. The gradient began with 86% A and 14% B, then changed to 70% B at minute 45. The flow rate was 0.15 mL/min. The column equilibration time was 15 min. The injection volume was 20 μL. HPLC was coupled to TOF/MS (time of flight mass spectrometry; Agilent 66230B; Yishun, Singapore) with an electrospray interface. The operating conditions were a gas temperature at 300 °C, gas flow at 6 L/min, nebulizer pressure at 60 PSIG, shredder at 200 V, skimmer at 65 V, and OCT RF Vpp at 750 V. Data were acquired using a Mass Hunter Workstation LC/MS data acquisition device with 6200 series software version B.06.01, build 6.0.633.10 (2012, Agilent Technologies, Santa Clara, CA, USA). The presence of each flavonoid was determined using its molecular ion derivatized with a H + (M + 1 uma), and compounds were identified following the methods of Pérez Gutiérrez et al. [26] and Son et al. [27].

### 4.2. Experimental Conditions—Animals

To minimize animal sacrifice and follow ethical standards regarding laboratory animals, we used rats that had been studied previously [65]. The highlighter extraction of polar components from the SME was carried out according to the protocol established by Pérez Gutiérrez et al. [26]. This extraction revealed a significant concentration of total polyphenols and demonstrated notable antioxidant activity. Additionally, it identified ten glucopyranose flavonoids sourced from the same farm field during the winter season. The sagittal sections of the kidneys were preserved immediately at −80 °C and stored in neutral formalin for biochemical and histological studies. Urine samples were collected under a layer of toluene to prevent bacterial growth and then stored at 4 °C until analysis.

### 4.3. Experimental Design of a Diabetes-Induced Kidney Damage Model in Rats and Treatments

This study was approved by the Research Committee of Juárez Hospital of Mexico (HJM/0713/19-I) and the Research Ethics Committee for the care and use of experimental animals of the National School of Biological Sciences of the IPN (ENCB/CEI/043/2019). Male Wistar rats (280 ± 10 g) were fasted for eight hours before hyperglycemia induction. The animals received a single intraperitoneal injection of streptozotocin (Sigma-Aldrich, St. Louis, MO, USA, Lot#119K1591) at a dose of 60 mg/kg body weight. The streptozotocin solution (100 mg/mL) was prepared in a 0.1 M citrate buffer (pH 4.5) consisting of 1.05 g of citric acid and 1.48 g of sodium citrate dissolved in 100 mL of distilled water. Three days post-injection, blood glucose levels were measured using a glucometer (One Touch Ultra Mini or ACCU-CHEK Active; Roche Diagnostics GmbH, Mannheim, Germany), with blood samples collected from the tail tip. Rats with blood glucose levels exceeding 350 mg/dL were included in the study. The ARRIVE Guidelines Checklist can be found in Supplementary Materials: Table S1.

The animals with STZ-induced hyperglycemia (STZ) were divided into two groups: STZ treated with vehicle (drinking water) in a volume of 1 mL/kg body weight and administered by oral gavage (STZ; *n* = 8) and STZ treated with 400 mg/kg of SME suspended in the vehicle (STZ-SME; *n* = 8). Additionally, rats with normal glycemia (CTLR; *n* = 8) and the CTLR treated with SME (SME; *n* = 8) were included. The assigned doses were administered daily for 12 weeks, and fasting glucose was measured weekly. Twelve weeks was selected because, at this time, there is significant accumulation of AGEs and morphological changes in the kidneys, reflecting progressive but still reversible renal damage, providing an appropriate window to assess the impact of SME before the disease reaches its terminal stage [66,67,68]. The SME dosage regimen, euthanasia, and biological sample collection were performed as previously reported [65]. The sagittal sections of both kidneys were preserved immediately at −80 °C and in neutral formalin for biochemical and histological studies. The urine was collected under a layer of toluene to inhibit bacterial growth and then stored at 4 °C until analysis.

### 4.4. Lipid Peroxidation

The kidney tissue underwent the lipid peroxidation assay using thiobarbituric acid reactive substances (TBARSs) following the manufacturer’s instructions (OXItek-TBARS assay kit, Enzo Life Sciences, Farmingdale, NY, USA). According to procedures described by Flores-Estrada et al. [65], MDA concentration (nmol/mg protein) was measured in the supernatant by absorbance at 532 nm (EON, BioTek Instruments, Inc., Winooski, VT, USA).

### 4.5. Detection of Total AGEs in Serum

Twelve weeks after the experiment, the rats were anesthetized with a ketamine–xylazine mixture. Blood samples (3.0 mL) were collected via the abdominal cava vein into a serum separator tube. The serum was clotted overnight at 4 °C and then centrifuged at 1000× *g* for 20 min and stored at −80 °C.

Serum levels of total AGEs were determined by using a Human Advanced Glycation Products (AGEs) ELISA kit (MBS267540; MyBiosource, Inc., San Diego, CA, USA). The serum and reaction mix were added to a 96-well plate and incubated at room temperature or 37 °C for 30 min. The serum concentration of AGEs was measured using a microplate reader (EON, BioTek Instruments, Inc., Winooski, VT, USA) at 450 nm with value correction. The concentrations were determined according to the manufacturer’s standard, and the AGEs (ηg/mL) were adjusted using the values of the CTRL group as a reference point for normalization.

### 4.6. Histology and Immunohistochemical (IHC) Staining

The kidney tissues were fixed in neutral formalin (10% formaldehyde in phosphate buffer solution (PBS, pH 7.4), dehydrated in graded alcohols (PBS-ethanol-Xylol), and embedded in paraffin. The tissue was cut into three-micron serial sections, mounted on electrostatic slides (CristalCruz, Santa Cruz Biotechnology, Inc., Dallas, TX, USA), and hydrated in graded alcohols (Xylol-ethanol-PBS). Hematoxylin-eosin (H&E) staining was used to assess inflammatory cell infiltration, which was scored as absent (0), mild (1), mild-moderate (2), moderate (3), or severe (4). Picrosirius red stain (PSR; ab150681; Abcam PLC, Cambridge, UK) was used to visualize collagen fiber deposition and determine the percentage of interstitial collagen (picture at 20× magnification). The percentage of collagen deposits was quantified using bright field microscopy at 20× magnification (Axio Imager.A2 microscope, Carl Zeiss Microscopy GmbH, Jena, Germany). This was carried out by calculating the total area of the picrosirius red (PSR) staining ratio. Eighty non-overlapping fields were randomly selected from each group of animals (*n =* 8), with ten fields analyzed for each animal. Two observers conducted pathological scoring independently.

For immunohistochemistry studies, tissue sections were deparaffinized, rehydrated, and subjected to antigen retrieval in 0.1 M citrate buffer (pH 6.0) using a pressure cooker. The slides were mounted in a Shandon Sequenza chamber (Thermo Fisher Scientific, Inc., Waltham, MA, USA) and incubated with blocking universal reagent (PolyVue^®^ mouse/rabbit DAB detection system, Diagnostic BioSystems, Pleasanton, CA, USA) at room temperature for one hour. Immediately, Primary antibody dilutions 1:100 for anti-CML (ab124145; Abcam PLC, Cambridge, UK), RAGE (sc-7269; Santa Cruz Biotechnology, Inc., Dallas, TX, USA), NF-κBp65 (sc-8008; Santa Cruz Biotechnology, Inc., Dallas, TX, USA), TNF-α (ab1793), IL-1β (sc-12742), casp-3 (sc-1224; Santa Cruz Biotechnology, Inc., Dallas, TX, USA), SMAD2/3 (ab63399), TGF-β1 (sc-31609), α-SMA (sc-53142; Santa Cruz Biotechnology, Inc., Dallas, TX, USA), CTGF (sc-365970; Santa Cruz Biotechnology, Inc., Dallas, TX, USA), and NOX4 (ab133303, Abcam PLC, Cambridge, UK) were added and incubated overnight at 4 °C. Later, secondary antibodies, anti-mouse, anti-rabbit and anti-goat IgG-HRP, were used and incubated for one hour at room temperature. 3,3’-diaminobenzidine was used as a chromogen substrate, and the slides were counterstained with Harris hematoxylin. To assess the staining intensity, histological images captured using Zen software version 12 (Carl Zeiss Microscopy GmbH, Jena, Germany) were used to measure staining intensity using Image-Pro-Plus software version 6.0 (Media Cybernetics, Inc., Rockville, MD, USA).

### 4.7. Immunofluorescence for CML and RAGE in Kidney Tissue

The double immunofluorescence staining for CML and RAGE was realized in the same slides, which were previously incubated with the blocking reagent solution (Diagnostic BioSystems, Pleasanton, CA, USA) for 60 min at room temperature. Then, the slides were incubated overnight at 4 °C with a mouse anti-CML antibody (1:100; ab124145; Abcam PLC, Cambridge, UK), followed by an Alexa 647-goat anti-rabbit IgG (1:100) at room temperature for 60 min. Afterward, the tissues were incubated with a rabbit anti-RAGE antibody (1:100; sc-365154) and an Alexa 594-goat anti-rabbit IgG (1:100) for 60 min. After washing with PBS, the sections were mounted with a medium containing 4′,6-Diamidine-2′-phenylindole dihydrochloride (DAPI) to visualize the cell nuclei. The fluorescence images were acquired using a FLoid™ Cell Imaging Station (Life Technologies Carlsbad, San Diego, CA, USA), and immunofluorescence staining analyses were performed with Image-Pro-Plus software version 6.0 (Media Cybernetics, Inc., Rockville, MD, USA). As a negative control, primary antibodies were excluded, and no specific immunostaining was observed.

### 4.8. Statistical Analysis

The staining intensity results from the immunohistochemical analyses were evaluated using integrated optical density (IOD), measured in lumens per pixel squared (lum/pixel^2^). This value was calculated by multiplying the total area by the mean density, utilizing Image-Pro Plus software version 6.0 (Media Cybernetics, Inc., Rockville, MD, USA). The percentage of cells with positive nuclei for NF-κB and SMAD2/3 in glomerular, tubular, and interstitial cells was quantified separately. Additionally, the cytoplasmic quantification of casp-3 staining in these cells was performed. The percentage was calculated as the ratio of stained cells (either nuclei or cytoplasm) to the total number of cells per field. Two observers independently captured the data. The fields were randomly chosen using a microscope with magnifications of 100× and 200×. At least 80 fields per group (*n* = 8; 10 fields for each animal) were utilized. The Kolmogorov–Smirnov test was used to determine the data distribution, resulting in a non-parametric distribution. In all cases, the Kruskal–Wallis ANOVA test was followed by the Dunnett multiple comparison test using GraphPad Prism software (La Jolla, CA, USA; version 8.0). The results were considered statistically significant when *p* < 0.05.

## 5. Conclusions

SME contains glycoside flavonoids that help reduce kidney damage in rats by decreasing acute tubular necrosis and glomerulopathy. This effect may be linked to its antioxidant properties and AGEs/RAGE axis attenuation, which reduces inflammation and fibrosis. Thus, spinach extract could serve as an alternative and nephroprotective treatment against diabetic kidney disease. Further investigation in clinical trials is necessary.

## Figures and Tables

**Table 1:** Characterization of flavonoids from SME using HPLC-ESI-TOF-MS.

Peak	Retention Time (t_R_; min)	Ion (*m*/*z*)	Compound	References
1	12.85	789	patuletin-3-O-β-d-glucopyranosyl-(1→6)-[β-d-apiofuranosyl-(1→2)]-β-d-glucopyranoside	[26]
2	18.38	803.25	spinacetin 3-O-glucosyl-(1→6)- [apiosyl(1→2)]-glucoside	[26,27]
3	21.28	935	patuletin-3-O-(2”-coumaroylglucosyl)-(1→6)-[apiosyl-(1→2)]-β-D-glucopyranoside	[26]
4	22.94	671.25	spinacetin-3-O-glucosyl-(1→6)-glucoside	[27]
5	34.59	817	spinacetin-3-O-(2”-coumaroylglucosyl)-(1→6)-β-D-glucopyranoside	[26]
6	35.39	847	spinacetin 3-O-(2”-feruloylglucosyl)-(1→6)-β-D glucopyranoside	[26]
7	37.61	523.15	spinatoside	[27]
8	38.82	537.15	jaceidin 4′-glucuronide	[27]

## Data Availability

The authors confirm that the data supporting the findings of this study are available within the article. Raw data are available from the corresponding author upon reasonable request.

## Supplementary Materials

The following supporting information can be downloaded at: https://www.mdpi.com/article/10.3390/ijms26104730/s1.

## References

[1] Akhtar M., Taha N.M., Nauman A., Mujeeb I.B., Al-Nabet A.D.M.H. (2020). Diabetic Kidney Disease: Past and Present. Adv. Anat. Pathol..

[2] Jung C.Y., Yoo T.H. (2022). Pathophysiologic Mechanisms and Potential Biomarkers in Diabetic Kidney Disease. Diabetes Metab. J..

[3] Cao Y., Lin J.H., Hammes H.P., Zhang C. (2022). Cellular Phenotypic Transitions in Diabetic Nephropathy: An Update. Front. Pharmacol..

[4] Wang Y., Jin M., Cheng C.K., Li Q. (2023). Tubular Injury in Diabetic Kidney Disease: Molecular Mechanisms and Potential Therapeutic Perspectives. Front. Endocrinol..

[5] Yamagishi S., Fukami K., Ueda S., Okuda S. (2007). Molecular Mechanisms of Diabetic Nephropathy and Its Therapeutic Intervention. Curr. Drug Targets.

[6] Samsu N. (2021). Diabetic Nephropathy: Challenges in Pathogenesis, Diagnosis, and Treatment. BioMed Res. Int..

[7] Fotheringham A.K., Gallo L.A., Borg D.J., Forbes J.M. (2022). Advanced Glycation End Products (AGEs) and Chronic Kidney Disease: Does the Modern Diet AGE the Kidney?. Nutrients.

[8] Miyata T., Ueda Y., Horie K., Nangaku M., Tanaka S., Van Ypersele De Strihou C., Kurokawa K. (1998). Renal Catabolism of Advanced Glycation End Products: The Fate of Pentosidine. Kidney Int..

[9] Vlassara H., Uribarri J., Cai W., Striker G. (2008). Advanced Glycation End Product Homeostasis: Exogenous Oxidants and Innate Defenses. Ann. N. Y. Acad. Sci..

[10] Pasupulati A.K., Nagati V., Paturi A.S.V., Reddy G.B. (2024). Non-Enzymatic Glycation and Diabetic Kidney Disease. Vitam. Horm..

[11] Tanji N., Markowitz G.S., Fu C., Kislinger T., Taguchi A., Pischetsrieder M., Stern D., Schmidt A.M., D’Agati V.D. (2000). Expression of Advanced Glycation End Products and Their Cellular Receptor RAGE in Diabetic Nephropathy and Nondiabetic Renal Disease. J. Am. Soc. Nephrol..

[12] Lu C., He J.C., Cai W., Liu H.I., Zhu L., Vlassara H. (2004). Advanced Glycation Endproduct (AGE) Receptor 1 Is a Negative Regulator of the Inflammatory Response to AGE in Mesangial Cells. Proc. Natl. Acad. Sci. USA.

[13] Shen C.Y., Lu C.H., Wu C.H., Li K.J., Kuo Y.M., Hsieh S.C., Yu C.L. (2020). The Development of Maillard Reaction, and Advanced Glycation End Product (AGE)-Receptor for AGE (RAGE) Signaling Inhibitors as Novel Therapeutic Strategies for Patients with AGE-Related Diseases. Molecules.

[14] Li J.H., Huang X.R., Zhu H.J., Oldfield M., Cooper M., Truong L.D., Johnson R.J., Lan H.Y. (2004). Advanced Glycation End Products Activate Smad Signaling via TGF-Beta-Dependent and Independent Mechanisms: Implications for Diabetic Renal and Vascular Disease. FASEB J..

[15] Oldfield M.D., Bach L.A., Forbes J.M., Nikolic-Paterson D., McRobert A., Thallas V., Atkins R.C., Osicka T., Jerums G., Cooper M.E. (2001). Advanced Glycation End Products Cause Epithelial-Myofibroblast Transdifferentiation via the Receptor for Advanced Glycation End Products (RAGE). J. Clin. Investig..

[16] Li J.H., Wang W., Huang X.R., Oldfield M., Schmidt A.M., Cooper M.E., Lan H.Y. (2004). Advanced Glycation End Products Induce Tubular Epithelial-Myofibroblast Transition through the RAGE-ERK1/2 MAP Kinase Signaling Pathway. Am. J. Pathol..

[17] Abdelkader H., Mustafa W.W., Alqahtani A.M., Alsharani S., Al Fatease A., Alany R.G. (2022). Glycation-Induced Age-Related Illnesses, Antiglycation and Drug Delivery Strategies. J. Pharm. Pharmacol..

[18] Pandjaitan N., Howard L.R., Morelock T., Gil M.I. (2005). Antioxidant Capacity and Phenolic Content of Spinach as Affected by Genetics and Maturation. J. Agric. Food Chem..

[19] Jaime L., Vázquez E., Fornari T., López-Hazas M.d.C., García-Risco M.R., Santoyo S., Reglero G. (2015). Extraction of Functional Ingredients from Spinach (*Spinacia oleracea* L.) Using Liquid Solvent and Supercritical CO_2_ Extraction. J. Sci. Food Agric..

[20] Dabeek W.M., Marra M.V. (2019). Dietary Quercetin and Kaempferol: Bioavailability and Potential Cardiovascular-Related Bioactivity in Humans. Nutrients.

[21] Roberts J.L., Moreau R. (2016). Functional Properties of Spinach (*Spinacia oleracea* L.) Phytochemicals and Bioactives. Food Funct..

[22] Bhuiyan M.N.I., Mitsuhashi S., Sigetomi K., Ubukata M. (2017). Quercetin Inhibits Advanced Glycation End Product Formation via Chelating Metal Ions, Trapping Methylglyoxal, and Trapping Reactive Oxygen Species. Biosci. Biotechnol. Biochem..

[23] Wang Y., Kim H.J., Sparrow J.R. (2017). Quercetin and Cyanidin-3-Glucoside Protect against Photooxidation and Photodegradation of A2E in Retinal Pigment Epithelial Cells. Exp. Eye Res..

[24] Bodiga V.L., Eda S.R., Veduruvalasa V.D., Mididodla L.D., Parise P.K., Kodamanchili S., Jallepalli S., Inapurapu S.P., Neerukonda M., Vemuri P.K. (2013). Attenuation of Non-Enzymatic Thermal Glycation of Bovine Serum Albumin (BSA) Using β-Carotene. Int. J. Biol. Macromol..

[25] Bautista-Pérez R., Cano-Martínez A., Gutiérrez-Velázquez E., Martínez-Rosas M., Pérez-Gutiérrez R.M., Jiménez-Gómez F., Flores-Estrada J. (2021). Spinach Methanolic Extract Attenuates the Retinal Degeneration in Diabetic Rats. Antioxidants.

[26] Perez Gutierrez R.M., Velazquez E.G. (2020). Glucopyranoside Flavonoids Isolated from Leaves of *Spinacia oleracea* (Spinach) Inhibit the Formation of Advanced Glycation End Products (AGEs) and Aldose Reductase Activity (RLAR). Biomed. Pharmacother..

[27] Son Y.G., Jung J., Lee D.K., Park S.W., Kim J.Y., Kim H.J. (2024). Validation of an Analytical Method of 3′,4′,5-Trihydroxy-3-Methoxy-6,7-Methylenedioxyflavone 4′-Glucuronide for Standardization of *Spinacia oleracea*. Molecules.

[28] Hashemzaei M., Tabrizian K., Alizadeh Z., Pasandideh S., Rezaee R., Mamoulakis C., Tsatsakis A., Skaperda Z., Kouretas D., Shahraki J. (2020). Resveratrol, Curcumin and Gallic Acid Attenuate Glyoxal-Induced Damage to Rat Renal Cells. Toxicol. Rep..

[29] Liu D., Chen J., Xie Y., Mei X., Xu C., Liu J., Cao X. (2022). Investigating the Molecular Mechanisms of Glyoxal-Induced Cytotoxicity in Human Embryonic Kidney Cells: Insights from Network Toxicology and Cell Biology Experiments. Environ. Toxicol..

[30] Nahdi A.M.T.A., John A., Raza H. (2017). Elucidation of Molecular Mechanisms of Streptozotocin-Induced Oxidative Stress, Apoptosis, and Mitochondrial Dysfunction in Rin-5F Pancreatic β-Cells. Oxidative Med. Cell. Longev..

[31] Nakai K., Umehara M., Minamida A., Yamauchi-Sawada H., Sunahara Y., Matoba Y., Okuno-Ozeki N., Nakamura I., Nakata T., Yagi-Tomita A. (2023). Streptozotocin Induces Renal Proximal Tubular Injury through P53 Signaling Activation. Sci. Rep..

[32] Naz R., Saqib F., Awadallah S., Wahid M., Latif M.F., Iqbal I., Mubarak M.S. (2023). Food Polyphenols and Type II Diabetes Mellitus: Pharmacology and Mechanisms. Molecules.

[33] Zhao P., Alam M.B., Lee S.H., Kim Y.J., Lee S., An H., Choi H.J., Son H.U., Park C.H., Kim H.H. (2017). Spatholobus Suberectus Exhibits Antidiabetic Activity In Vitro and In Vivo through Activation of AKT-AMPK Pathway. Evid.-Based Complement. Alternat. Med..

[34] Do M.H., Hur J., Choi J., Kim Y., Park H.Y., Ha S.K. (2018). Spatholobus Suberectus Ameliorates Diabetes-Induced Renal Damage by Suppressing Advanced Glycation End Products in Db/Db Mice. Int. J. Mol. Sci..

[35] Yuan Y., Sun H., Sun Z. (2017). Advanced Glycation End Products (AGEs) Increase Renal Lipid Accumulation: A Pathogenic Factor of Diabetic Nephropathy (DN). Lipids Health Dis..

[36] Nerlich A.G., Schleicher E.D. (1999). N(Epsilon)-(Carboxymethyl)Lysine in Atherosclerotic Vascular Lesions as a Marker for Local Oxidative Stress. Atherosclerosis.

[37] Teissier T., Quersin V., Gnemmi V., Daroux M., Howsam M., Delguste F., Lemoine C., Fradin C., Schmidt A., Cauffiez C. (2019). Knockout of Receptor for Advanced Glycation End-products Attenuates Age-related Renal Lesions. Aging Cell.

[38] Rabbani N., Thornalley P.J. (2018). Advanced Glycation End Products in the Pathogenesis of Chronic Kidney Disease. Kidney Int..

[39] Wagner Z., Wittmann I., Mazak I., Schinzel R., Heidland A., Kientsch-Engel R., Nagy J. (2001). Nε-(Carboxymethyl)Lysine Levels in Patients with Type 2 Diabetes: Role of Renal Function. Am. J. Kidney Dis..

[40] Maessen D.E.M., Stehouwer C.D.A., Schalkwijk C.G. (2015). The Role of Methylglyoxal and the Glyoxalase System in Diabetes and Other Age-Related Diseases. Clin. Sci..

[41] Vistoli G., De Maddis D., Cipak A., Zarkovic N., Carini M., Aldini G. (2013). Advanced Glycoxidation and Lipoxidation End Products (AGEs and ALEs): An Overview of Their Mechanisms of Formation. Free Radic. Res..

[42] Asano M., Fujita Y., Ueda Y., Suzuki D., Miyata T., Sakai H., Saito A. (2002). Renal Proximal Tubular Metabolism of Protein-Linked Pentosidine, an Advanced Glycation End Product. Nephron.

[43] Miyata T., Van Ypersele de Strihou C., Imasawa T., Yoshino A., Ueda Y., Ogura H., Kominami K., Onogi H., Inagi R., Nangaku M. (2001). Glyoxalase I Deficiency Is Associated with an Unusual Level of Advanced Glycation End Products in a Hemodialysis Patient. Kidney Int..

[44] Curran C.S., Kopp J.B. (2022). RAGE Pathway Activation and Function in Chronic Kidney Disease and COVID-19. Front. Med..

[45] Kierdorf K., Fritz G. (2013). RAGE Regulation and Signaling in Inflammation and Beyond. J. Leukoc. Biol..

[46] Sun H., Yuan Y., Sun Z. (2016). Update on Mechanisms of Renal Tubule Injury Caused by Advanced Glycation End Products. Biomed Res. Int..

[47] He C.-J., Zheng F., Stitt A., Striker L., Hattori M., Vlassara H. (2000). Differential Expression of Renal AGE-Receptor Genes in NOD Mice: Possible Role in Nonobese Diabetic Renal Disease. Kidney Int..

[48] Liu J., Huang K., Cai G.Y., Chen X.M., Yang J.R., Lin L.R., Yang J., Huo B.G., Zhan J., He Y.N. (2014). Receptor for Advanced Glycation End-Products Promotes Premature Senescence of Proximal Tubular Epithelial Cells via Activation of Endoplasmic Reticulum Stress-Dependent P21 Signaling. Cell. Signal..

[49] Cheng M., Liu H., Zhang D., Liu Y., Wang C., Liu F., Chen J. (2015). HMGB1 Enhances the AGE-Induced Expression of CTGF and TGF-β via RAGE-Dependent Signaling in Renal Tubular Epithelial Cells. Am. J. Nephrol..

[50] Penfold S.A., Coughlan M.T., Patel S.K., Srivastava P.M., Sourris K.C., Steer D., Webster D.E., Thomas M.C., MacIsaac R.J., Jerums G. (2010). Circulating High-Molecular-Weight RAGE Ligands Activate Pathways Implicated in the Development of Diabetic Nephropathy. Kidney Int..

[51] You X.L., Zhao M.L., Liu Y.R., Tang Z.S., Zhao Y.T., Yan-Liu, Song Z.X. (2024). *Hypericum perforatum* L. Protects against Renal Function Decline in Ovariectomy Rat Model by Regulating Expressions of NOS3 and AKT1 in AGE-RAGE Pathway. Phytomedicine.

[52] Kim Y.S., Jung D.H., Lee I.S., Pyun B.J., Kim J.S. (2016). Osteomeles Schwerinae Extracts Inhibits the Binding to Receptors of Advanced Glycation End Products and TGF-Β1 Expression in Mesangial Cells under Diabetic Conditions. Phytomedicine.

[53] Kim Y.S., Jung D.H., Lee I.S., Choi S.J., Yu S.Y., Ku S.K., Kim M.H., Kim J.S. (2013). Effects of *Allium victorialis* Leaf Extracts and Its Single Compounds on Aldose Reductase, Advanced Glycation End Products and TGF-Β1 Expression in Mesangial Cells. BMC Complement. Altern. Med..

[54] Li X., Xiao Y., Gao H., Li B., Xu L., Cheng M., Jiang B., Ma Y. (2009). Grape Seed Proanthocyanidins Ameliorate Diabetic Nephropathy via Modulation of Levels of AGE, RAGE and CTGF. Nephron Exp. Nephrol..

[55] Hou B., Qiang G., Zhao Y., Yang X., Chen X., Yan Y., Wang X., Liu C., Zhang L., Du G. (2017). Salvianolic Acid A Protects Against Diabetic Nephropathy through Ameliorating Glomerular Endothelial Dysfunction via Inhibiting AGE-RAGE Signaling. Cell. Physiol. Biochem..

[56] Qiu Y.Y., Tang L.Q., Wei W. (2017). Berberine Exerts Renoprotective Effects by Regulating the AGEs-RAGE Signaling Pathway in Mesangial Cells during Diabetic Nephropathy. Mol. Cell. Endocrinol..

[57] Mou X., Zhou D.Y., Zhou D., Liu K., Chen L.J., Liu W.H. (2020). A Bioinformatics and Network Pharmacology Approach to the Mechanisms of Action of Shenxiao Decoction for the Treatment of Diabetic Nephropathy. Phytomedicine.

[58] Niu H., Fan L., Zhao L., Yao R., He X., Lu B., Pang Z. (2022). The Therapeutic Mechanism of PuRenDan for the Treatment of Diabetic Nephropathy: Network Pharmacology and Experimental Verification. J. Ethnopharmacol..

[59] Chen Y., Chen J., Jiang M., Fu Y., Zhu Y., Jiao N., Liu L., Du Q., Wu H., Xu H. (2020). Loganin and Catalpol Exert Cooperative Ameliorating Effects on Podocyte Apoptosis upon Diabetic Nephropathy by Targeting AGEs-RAGE Signaling. Life Sci..

[60] Liu C., Zhu R., Liu H., Li L., Chen B., Jia Q., Wang L., Ma R., Tian S., Wang M. (2018). Aqueous Extract of Mori Folium Exerts Bone Protective Effect Through Regulation of Calcium and Redox Homeostasis via PTH/VDR/CaBP and AGEs/RAGE/Nox4/NF-ΚB Signaling in Diabetic Rats. Front. Pharmacol..

[61] Park C.H., Yokozawa T., Noh J.S. (2014). Oligonol, a Low-Molecular-Weight Polyphenol Derived from Lychee Fruit, Attenuates Diabetes-Induced Renal Damage through the Advanced Glycation End Product-Related Pathway in Db/Db Mice. J. Nutr..

[62] Jha J.C., Gray S.P., Barit D., Okabe J., El-Osta A., Namikoshi T., Thallas-Bonke V., Wingler K., Szyndralewiez C., Heitz F. (2014). Genetic Targeting or Pharmacologic Inhibition of NADPH Oxidase Nox4 Provides Renoprotection in Long-Term Diabetic Nephropathy. J. Am. Soc. Nephrol..

[63] de Araújo E.R.D., Guerra G.C.B., de Souza Araújo D.F., de Araújo A.A., Fernandes J.M., de Araújo Júnior R.F., da Silva V.C., de Carvalho T.G., de Santis Ferreira L., Zucolotto S.M. (2018). Gastroprotective and Antioxidant Activity of *Kalanchoe brasiliensis* and *Kalanchoe pinnata* Leaf Juices against Indomethacin and Ethanol-Induced Gastric Lesions in Rats. Int. J. Mol. Sci..

[64] Fukami K., Ueda S., Yamagishi S.I., Kato S., Inagaki Y., Takeuchi M., Motomiya Y., Bucala R., Iida S., Tamaki K. (2004). AGEs Activate Mesangial TGF-Beta-Smad Signaling via an Angiotensin II Type I Receptor Interaction. Kidney Int..

[65] Flores-Estrada J., Cano-Martínez A., Vargas-González Á., Castrejón-Téllez V., Cornejo-Garrido J., Martínez-Rosas M., Guarner-Lans V., Rubio-Ruíz M.E. (2023). Hepatoprotective Mechanisms Induced by Spinach Methanolic Extract in Rats with Hyperglycemia-An Immunohistochemical Analysis. Antioxidants.

[66] Putt D.A., Zhong Q., Lash L.H. (2012). Adaptive Changes in Renal Mitochondrial Redox Status in Diabetic Nephropathy. Toxicol. Appl. Pharmacol..

[67] Mitsuhashi T., Nakayama H., Itoh T., Kuwajima S., Aoki S., Atsumi T., Koike T. (1993). Immunochemical Detection of Advanced Glycation End Products in Renal Cortex from STZ-Induced Diabetic Rat. Diabetes.

[68] Jozi F., Kheiripour N., Taheri M.A., Ardjmand A., Ghavipanjeh G., Nasehi Z., Shahaboddin M.E. (2022). L-Lysine Ameliorates Diabetic Nephropathy in Rats with Streptozotocin-Induced Diabetes Mellitus. BioMed Res. Int..

